# Assessment of the Effect of Multiple Processing of PHBV–Ground Buckwheat Hull Biocomposite on Its Functional and Mechanical Properties

**DOI:** 10.3390/ma17246136

**Published:** 2024-12-15

**Authors:** Grzegorz Janowski, Marta Wójcik, Wiesław Frącz, Łukasz Bąk, Grażyna Ryzińska

**Affiliations:** Department of Materials Forming and Processing, Rzeszow University of Technology, Powstańców Warszawy 8, 35-959 Rzeszów, Poland; wf@prz.edu.pl (W.F.); lbak@prz.edu.pl (Ł.B.); grar@prz.edu.pl (G.R.)

**Keywords:** natural fillers, biocomposites, buckwheat hulls, reprocessing, recycling, injection molding

## Abstract

The influence of the addition of ground buckwheat hulls on the properties of biocomposite on the basis of 3-hydroxybutyrate-co-3-hydroxyvalerate (PHBV) is presented here. The changes in the material after repeated reprocessing—up to five recycling cycles—are written in the paper. Analysis of the shrinkage, water adsorption, selected mechanical properties, tensile impact strength, hardness and the microstructure of the surface layer was performed. The results show that the application of the buckwheat hulls into the biopolymer decreases the material shrinkage. It improves the material dimensional stability, as well as increases the water adsorption in the wake of the hydrophobic properties of the filler. The addition of the natural filler also leads to an increase in composite stiffness. The decrease in the tensile impact strength and the elongation at break is also noted. The reprocessing of the biocomposite initially led to a decrease in its mechanical properties, but the results stabilized after further processing cycles. This indicates the improvement of the microstructure homogeneity. The microscopic analysis shows that buckwheat hull particles were better embedded in the matrix after recycling. The increase in hardness was also noted. The PHBV–ground buckwheat hull biocomposite is characterized by stable mechanical properties and by recycling resistance, which makes it a promising material in terms of the sustainable development.

## 1. Introduction

Nowadays, due to the increasing prices of synthetic polymers and the tightening of ecological regulations limiting their use, biopolymers are becoming increasingly important. Currently, biopolymers constitute less than 1% of total polymer production [1], which approximately equates to 320 million tons every year [2]. The global worldwide production of biopolymers was about 2.44 million tons in 2022 [3]. It is expected that this amount will increase to 7.5 million tons in 2029 [4].

Biopolymers are biocompatible, bio-based and/or biodegradable materials obtained from bio-based and fossil-fuel-based resources [5]. The biodegradable polymers consist of 42.9% of the biopolymer production [6], among which the most popular is poly(lactic acid) (PLA) derived from different renewable sources [7]. It is estimated that PLA represents about 40% of the whole biodegradable polymers production [8].

The bio-based and non-biodegradable biopolymers are a dominant group of biopolymers that accounts for 57.1% of their production [6]. The ability to biodegrade and the quality of biocompatibility mean that biopolymers are considered sustainable and ecologically friendly materials that might be successfully applied in many sectors of the economy, e.g., in the packaging industry, which uses approximately 58% of the total volume share [9]. The increasing interest in biopolymers results from their favorable electronical properties, low thermal expansion coefficient and the relatively high chemical resistance [10,11,12]. However, biopolymers without any additions are characterized by low mechanical properties. They also indicate poor forming ability [13].

Different non-toxic organic and non-organic fillers, especially in the form of fibers, are added to biopolymers in order to improve their properties while reducing production costs in comparison to the pure biopolymer [14]. Clays and nanoclays [15,16,17], bioactive glass [18], silica [19] and natural rubber [20] are examples of fillers applied in the formation of biopolymer composites. The plant-based products and food waste are more and more often used as fillers. The plant-based by-products and waste are tested as filler in biopolymer composites, e.g., flax [21], sawdust [22], cotton [23,24], jute [25], wood flour [26], kenaf fibers [27], biochar [28], coffee grounds [29], rice husk [30,31,32] and egg shells [33]. There are known examples of works concerning the application of grape seeds [34], olive pit powder [35,36], starch [37], and potato pulp powder [38] as fillers in biopolymer composites. The plant-based fillers, mainly in the form of short and continuous fibers, are characterized by the low density, as well as by the high stiffness and strength [39]. Their addition to biopolymers often results in an increase in mechanical properties of biopolymer composites. In [40], the application of harakeke to PLA in amount of 30% wt. has resulted in an increase in mechanical properties in comparison to the pure biopolymer. The increase in the elastic modulus by 9.18% and the yield strength by 54.43% for the biopolymer composite with spent coffee grounds in amount of 3% wt. compared with pure PLA was noted in [41]. The increase in mechanical properties for PLA and rice residue biopolymer composite was also indicated by Sun et al. [42].

The increasing interest in biopolymers is also caused by their recyclability [43]. Although the biopolymer prices are two to five times higher than conventional polymers [44,45], their utilization cost is even six times lower than for synthetic plastics [46,47]. The recycling methods of biopolymers are as follows: the mechanical recycling including shredding, melting and if necessary washing and drying; the chemical recycling (hydrolysis, alcohollysis) and depolymerization in high temperatures in the presence of catalysts [48,49,50,51]. The biodegradable biopolymers can be decomposed in the wake of enzymatic work of microorganism into environmentally friendly materials, i.e., biomass, carbon dioxide, methane and water, under special conditions. Although the biodegradation is an environmentally friendly utilization method, the degradation time for biopolymers might be long. For example, PLA biodegrades longer than 180 days outdoors [48,49]. The mechanical recycling in which the recovered material might be applied in the production of new goods with the same or similar purpose as the origin, might be an alternative method.

The possibility of recycling and the decreasing of production costs have caused the development of the biopolymer composites market with natural fillers that are often wastes with utilization difficulties. In previous works of authors [52,53,54], different natural fillers were added to the biopolymer matrix in order to produce biopolymer composites. The grounded buckwheat hulls (GBHs) are used as a natural filler in this work. The buckwheat hulls are neutral and environmentally friendly and indicate oxidative and bacteriostatic properties. The examples of the application of GBHs for the production of different materials are known [55,56,57], but their use as a filler in biopolymer composites is tested here for the first time.

The aim of the paper is the application of GBHs as a natural filler to the poly(3-hydroxybutyrate-co-3-hydroxyvalerate) (PHBV) matrix and the evaluation of the selected properties of the biocomposite obtained in terms of the recycling process. The GBHs were added to the PHBV matrix in a dosage of 45% wt. The dry mixtures of PHBV and GBHs were extruded using the single-screw extruder in conjunction with the granulation process. The granulate obtained was then applied in the injection-molding process. The selected properties of ground buckwheat hull–PHBV (GBH–PHBV) samples, e.g., hardness, shrinkage, mechanical properties and the change of the microstructure on the surface layer, were examined in the context of mechanical recycling possibilities by processing the material five times. The results show that the addition of the GBHs as the filler influences the mechanical and forming properties of the biocomposite in a significant way. The GBH–PHBV biocomposite is characterized by the lower shrinkage in comparison to the pure PHBV. The increase in the water adsorption in the wake of the addition of GBHs was also noted. But the reduction in the parameter value after several processing cycles was observed in the wake of the better settlement of filler particles in the matrix. The analysis of results has also shown the increase in the biopolymer hardness in comparison to the pure PHBV. The hardness has also increased with each subsequent processing cycle. It also leads to the decrease in the elastic modulus and the tensile impact strength. The microstructure photographs show that the microstructure has also changed with each subsequent processing cycle. The results obtained and conclusions drawn from their analysis confirm the great potential of the material in terms of sustainable development and indicate the purposefulness of further research related to its application.

## 2. Materials and Methodology

### 2.1. Materials

The powdered PHBV polymer die with a trade name ENMAT Y1000 produced by Tianan Biopolymer Company (Ningbo, China) was used in this research. The ground buckwheat hulls (Bio Planet S.A., Leszno, Poland) with 45% wt. were used as the filler here.

### 2.2. Production, Forming and Recycling of the Biocomposite

The dry mixture of PHBV and GBHs was prepared manually in order to obtain as homogeneous a composition as possible. Because both GBHs and PHBV have hygroscopic properties and have the ability to form large agglomerates, which can affect the possibility of obtaining the homogeneous structure of the composite in a negative way, the mixture was dried before the single-screw extrusion. The materials were dried for 6 h at 90 °C using the DZ-2BC laboratory dryer (Chemland, Poland). This stage was conducted in order to reduce the air bubbles in the cross-sections of extrudates.

The GBH–PHBV biocomposites were extruded by the ZAMAK EHP-25E single-screw extruder (ZAMAK, Skawina, Poland) with constant process temperature values in particular heating zones. The temperatures used are as follows: the head of the extruder-170 °C, zone 1–145 °C, zone 2–162 °C, zone 3–165 °C, charge-32 °C. The constant screw rotation speed of 25 rpm was applied here. The granulation of the material was performed in parallel with the extrusion process using the ZAMAK G16/32-II granulator (ZAMAK, Skawina, Poland). It allows for the optimization of the extrusion process and for the preparation of granulate dedicated to the injection-molding one. The granulate prepared was dried for 6 h at 90 °C before the injection-molding process.

The DrBoy 55E injection-molding machine (Dr.Boy, Neustadt, Germany), equipped with a Priamus system for monitoring and controlling the injection-molding process, was used here. The injection mold with the insert dedicated to the production of samples using a uniaxial tensile test, i.e., paddle sample (in accordance with EN ISO 527-1 standard [58]), is applied in this research. The granulate produced during the single-screw extrusion process was injected in accordance with the adjustable parameters presented in Table 1.

For the molders prepared in the previous part of the research, the granulation was carried out by Wanner C17.26 grinding mill (Wanner, Wertheim-Reicholzheim, Germany), dedicated to plastics. The regranulate was then dried for 6 h at 90 °C.

Based on the adjustable parameters previously determined, the injection-molding tests were conducted. The granulate obtained from previously ground molders was injected with a lower melt viscosity and, therefore, the melt temperature was reduced to 170 °C. The adjustable parameters did not change in comparison to the injection of biocomposite originally produced. Five recycling cycles were examined here. The decrease in viscosity was not observed during subsequent cycles and, therefore, the melt temperature remained at the level of 170 °C. For both biocomposites originally produced, as well as for reprocessed ones, the paddle samples were prepared and were used for further research.

### 2.3. Methodology

The shrinkage of samples originally produced and reprocessed ones was assessed by the measurement of dimensions (length, width and thickness of paddle shapes) in comparison to the dimensions of the injection-mold cavity.

**Figure 1:**
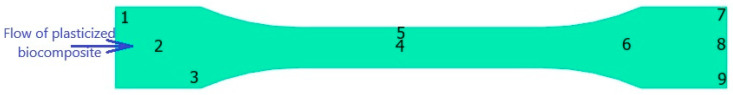
Specimen with marked places used for the determination of selected parameters ((1–9)–number of measuring point).

The structure of the surface layer was performed by Nikon MM-800 (Nikon Company, Tokio, Japan) microscope equipped with a digital camera. The photographs of surfaces at 30× magnification were made at precisely marked places of samples. The location of such places in samples was presented in Figure 1.

The Brinell hardness was carried out in line with EN ISO 2039-1 [59] standard by ZWICK 3106 hardness tester (ZWICK, Ulm, Germany). The hardness was measured in places presented in Figure 1.

The strength parameters were determined using Zwick Z030 testing machine (ZWICK, Germany). The uniaxial tensile tests were carried out for paddle samples in accordance with EN ISO 527-1 standard [58].

The impact tensile strength was determined in accordance with EN ISO 8256 standard [60]. The CAEST 9050 pendulum impact hammer (Instron, Norwood, MA, USA) was applied in this research. The specimens were prepared on the basis of paddle ones dedicated to the uniaxial tensile tests by the geometry modification in line with applicable standards.

The water absorption of samples was examined in accordance with EN ISO 62 [61] standard.

## 3. Results and Discussion

For the results presented below, the following signs are introduced:

- 0—the biocomposite originally produced;
- 1x, 2x, …, 5x—sets of biocomposite samples injected during the subsequent recycling cycles (1x—the first recycling cycle, 2x—the second recycling cycle, etc.);
- PHBV—pure poly(3-hydroxybutyrate-co-3-hydroxyvalerate). The properties were taken from [53,62].

**Figure 2:**
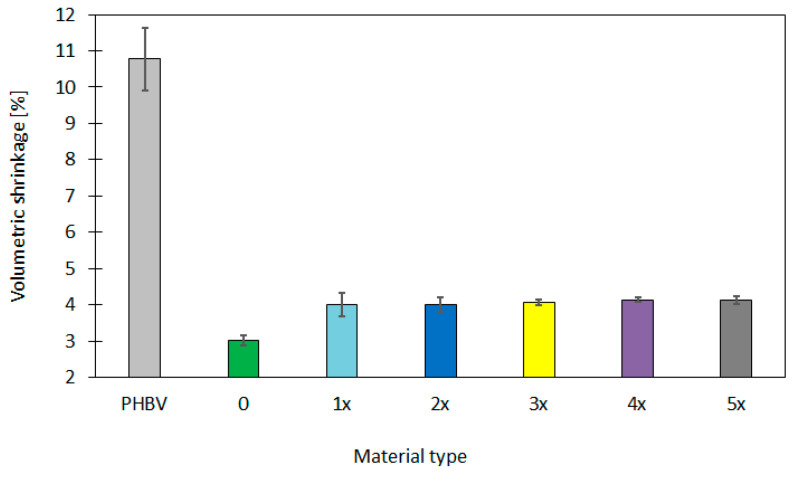
The volumetric shrinkage for biocomposites used in the research.

Firstly, the analysis of shrinkage was conducted. The longitudinal, transverse, volumetric and in-the-thickness shrinkage was assessed (Table 2, Figure 2).

It was noted that the average value of longitudinal shrinkage for the sample originally produced was 1.07%. After the first recycling cycle, it has increased to 1.22%. The longitudinal shrinkage was in the range of 1.21–1.23% for further cycles, which suggests the low impact of the recycling cycle on this parameter value. For the first processing cycle, the standard deviation increased from 0.0142% (“0” sample) to 0.0253% (1x). The decrease to the value of 0.0120% was noted for the third cycle (3x). The low variation coefficient indicates the low fluctuation in test results. The highest variation coefficient value was noted for the first recycling cycle (0.0208).

The transverse shrinkage for the “0” sample was 1.35% and incresed significantly to 1.91% after the first recycling cycle. Then, the transverse shrinkage was in the range of 1.90–1.96%, which can indicate the parameters’ stabilization after the first cycle. The standard deviation incresed to up 0.142% after the first recycling cycle, and then the decreasing trend was observed. This might suggest the stabilization of the process. The variation coefficient has also decresed during the research.

Shrinkage, in terms of thickness, was characterized by the lowest values of all the measured parameters. It was 0.62% for the “0” sample and increased to 0.93% after the first recycling cyle. The thickness shrinkage was 1.05% in the last tested cycle (5x). The standard deviation was the highest for the sample after the first recycling process (0.1983%), and then it decreased, which can indicate greater uncertainty in the results at the early stage of recycling. The variation coefficient reached the highest value for the “1x” sample (0.2128), and then it decreased, showing the improvement in the uniformity of results in subsequent cycles.

The volumetric shrinkage was 3.02% for the “0” sample and increased to 4.01% after the first recycling cycle. For further cycles, the parameter increased to a lesser extent and the volumetric shrinkage was 4.12% for the “5x” sample. The standard deviation increased after the first cycle. It then stabilized, which can indicate the higher certainty of results after several processing cycles. The variation coefficient had the highest value for the first recycling cycle (0.08) and then decreased. This might suggest the stabilization of the process. 

The pure PHBV biopolymer indicated higher shrinkage values in comparison to the biopolymer composite with the addition of GBHs, especially for the volumetric, longitudinal and in-the-thickness ones, which were as follows: 10.77%, 2.50% and 4.85%, respectively. The high shrinkage values for the pure PHBV biopolymer are a result of the high mobility of polymer chains during the cooling process, which leads to the significant material contraction. The lack of filler limited the deformation, meaning that the pure PHBV is more susceptible to the dimensional changes and is less dimensionally stable in the injection-molding process. This might influence the quality of samples [63,64].

As can be seen from the data obtained, the use of GBHs as a filler can influence the shrinkage significantly. It can be seen both for samples originally produced, as well as for ones obtained after the recycling process. The mineral fillers, e.g., the powdered buckwheat hulls, contribute to the reduction in polymer shrinkage due to the limitation of chain transition during the cooling process [65]. This leads to the smaller dimensional changes. The addition of GBHs can also improve the dimensional stability in subsequent recycling cycles because the powder structure contained in buckwheat hulls acts as a micro reinforcement that prevents the excessive shrinkage of the material [66,67]. The application of GBHs in biopolymers also stabilizes the material structure provided that the powder is evenly distributed in the polymer. The uneven distribution of the filler can contribute to the differences in shrinkage in particular parts of the molder.

It was noted that the recycling of GBH–PHBV biocomposites affects the shrinkage parameters significantly, especially for the first cycle. The analysis of shrinkage values after particular recycling cycles shows the stabilization of the structure after the first cycle and the improvement of material homogeneity in subsequent ones. Such observations are very important for the proper design of the recycling process of biopolymer composites, for which the control of shrinkage parameters is essential.

**Figure 3:**
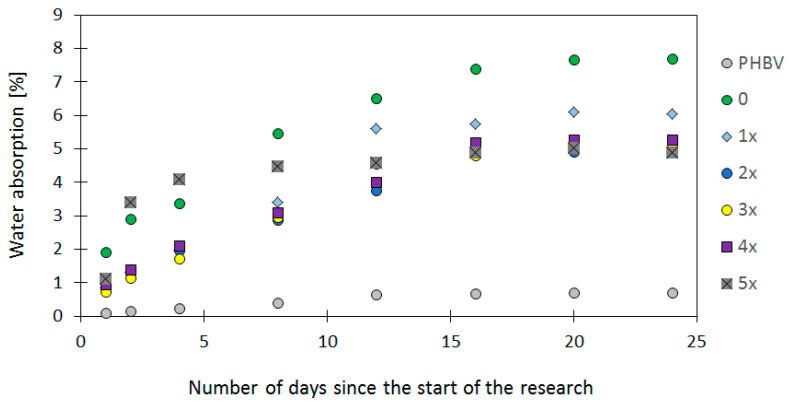
The water adsorption for GBH–PHBV biopolymer composites (samples 0–5x) and for the pure PHBV.

The analysis of the water adsorption for samples produced from pure PHBV and from PHBV in conjunction with GBHs shows the significant differences in the behavior of materials in contact with water (Figure 3). It is particularly visible in long-term tests.

The pure PHBV biopolymer indicated the low water adsorption from 0.097% after 1 day of immersion in water to up 0.704% after 24 days. The low water adsorption is characteristic for polymer materials with the high resistance to moisture. It is a result of the lack of hydrophilic functional groups in the PHBV structure. The literature review [46,47,48,49,50,51] emphasizes that pure PHBV is characterized by high hydrophobic properties, which means that the water adsorption is limited and the material indicates good mechanical properties even in the moist environment.

The GBH–PHBV biocomposite originally produced (“0” sample) indicated higher water adsorption in comparison to the pure PHBV. It was 1.904% after one day of immersion in water and increased to 7.688% after 24 days of the test. The increase in water adsorption is a result of the presence of buckwheat hulls in the polymer, which act as a natural hydrophilic filler. They also contain cellulose and lignin, which are characterized by the high ability to adsorb water. The main chemical groups responsible for the water adsorption of BH are hydroxyl and carboxyl ones. This leads to an increase in absorbency throughout the whole composite. The observations are consistent with results obtained by other researchers [51,52,53,54,55,56,57,58,59,60,61,62,63,64], who show that biocomposite polymers with natural fillers indicate higher water adsorption than their polymer equivalents.

For the first recycling cycle, the water adsorption was 0.835% after one day of immersion in water and increased to 6.036% after 24 days. In 2x–5x cycles, the parameter initially increased, reaching a maximum value for the “5x” sample (1.110% after one day and 4.903% after 24 days). The results suggest that recycling affects the microstructure of the biocomposite, leading to a decrease in the water absorption for recycling cycles.

In the literature [68,69], similar results are associated with the change in the polymer structure during the recycling process. The several processing can lead to better wetting and embedding of the filler in the polymer matrix. It also limits the availability of hydrophilic sites for water adsorption. The fine powder particles of BH are more uniformly embedded in the matrix after the subsequent recycling cycles. They might crease a barrier, which reduces the penetration of water into the material.

**Figure 4:**
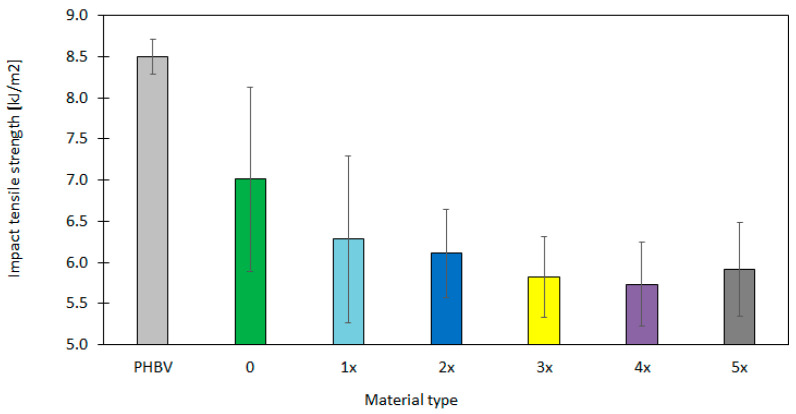
The graphical representation of the tensile impact strength for the biocomposite originally produced (“0” sample) and after the subsequent recycling cycles (1x–5x) in comparison to the pure PHBV.

The analysis of the tensile impact strength for both pure PHBV and GBH–PHBV biocomposites (originally produced and after the subsequent recycling cycles) shows the significant difference in behaviour of materials, which might be explained by the structure and the addition of the filler (Figure 4). The pure PHBV is characterized by the highest tensile impact strength at the level of approximately 8.5 kJ/m^2^ and by the low variability of results. This might indicate the ductile character of the material and its ability to dissipate the impact energy due to its flexible structure. In the literature [70,71], the PHBV biopolymer is considered a material with a good impact strength. This might be caused by the plastic deformation and the fracture propagation.

The application of powdered GBHs to the biocomposite originally produced leads to a reduction in the tensile impact strength to the value of 7.01 kJ/m^2^. On the other hand, the repeatability of properties is higher, which shows the higher variability in the wake of uneven distribution of fillers in the matrix. The presence of rigid filler particles acts as the potential crack initiation points, which limit the material ability to absorb energy. Similar effects are noted in [72,73], in which composites with natural fillers, especially in the powdered form, indicate the reduced impact strength due to the more brittle nature of the filler.

The analysis of the literature shows that the addition of natural fillers, e.g., buckwheat hulls, rice husk or cellulose fibers, can decrease the tensile impact strength, which is caused by their brittle and rigid nature [72,73,74]. Such fillers act as the crack initiators; therefore, the ability of the composite to dissipate energy during the impact is reduced. The results obtained for the GBH–PHBV biocomposite are consistent with the research carried out by other authors who have shown that powdered fillers with irregular shapes often caused the local stress concentration [72,73,74]. This can lead to earlier damage of the material. For samples after several processing cycles (1x–5x), the impact strength decreased gradually and reached the minimum value of 5.73 kJ/m^2^ for the fourth cycle. It increased slightly to the value of 5.92 kJ/m^2^ for the fifth cycle. The decrease in the tensile impact strength after the recycling process might be a result of the structural degradation of the polymer, as well as the fragmentation of the filler particles. This leads to the deterioration of adhesion between phases. However, the stabilization of results for further processing cycles might suggest the improvement of microstructure homogeneity in the wake of the better wetting of GBH particles by the polymer matrix.

**Figure 5:**
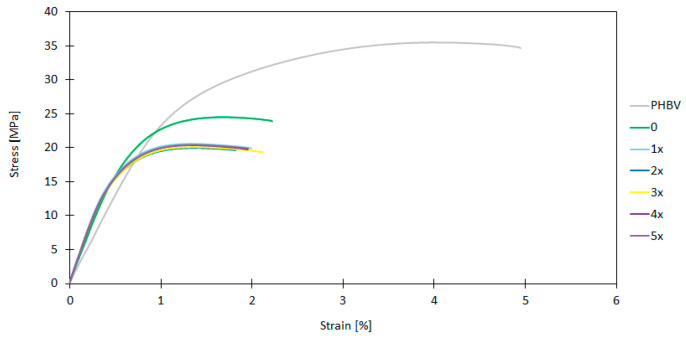
The stress–strain curves for the pure PHBV biopolymer and for the GBH–PHBV biocomposite (samples 0–5x).

The selected mechanical parameters of GBH–PHBV biocomposite were determined and analyzed on the basis of results obtained in uniaxial tension tests. The Young modulus (E), the tensile strenght (σ_M_) and the elongation at break (ε_M_) were examined here. The results for both originally produced samples and for ones after subsequent recycling cycles are shown in Table 3. The stress–strain curves for all samples were shown in Figure 5.

The significant difference was noted between the pure PHBV biopolymer and GBH–PHBV biocomposites for the Young elastic modulus (see Table 3). The Young modulus for the pure PHBV biopolymer was 2617.37 MPa. The “0” sample was characterized by the much higher value of the elastic modulus (3620.52 MPa), which is the result of the stiffening of the biocomposite by the addition of small powdered particles that act as a reinforcement. During the subsequent processing cycles, the elastic modulus increased to 4147.43 MPa for the fifth cycle. The increase in the parameter might be a result of a more uniform distribution of GBHs.

The tensile strength of the pure PHBV biopolymer was 35.48 MPa, and the decrease to 24.50 MPa for the “0” sample was noted. This might indicate that the reduction is caused by the distribution of particles, which can lead to local stress concentrations. Although after the first recycling cycle (1x) the tensile strength decreased to 20.57 Mpa, for further cycles this parameter stabilized at the level of 19.96–20.57 MPa. The decrease might be related to the change of biopolymer structure during the recycling process. At the same time, the better embedding of powder particles results in relatively stable values for tensile strength.

The analysis of the elongation at break shows the significant differences between PHBV and biopolymer composites (see Table 3). For the pure PHBV biopolymer, the ε_M_ parameter was 4.12%, and it was much higher than for other samples. The elongation at break for the “0” sample was 1.70%, which suggests that the material is very brittle after the addition of buckwheat hulls into the biopolymer matrix. During the further recycling cycles, this parameter has a relatively stable value in the range of 1.30–1.37%. The decrease in the elongation for biocomposites after the application of GBHs as a filler might be caused by the reduction in the mobility of polymer chains due to the presence of rigid particles in them. The stress–strain curves also confirm these observations. The highest elongation was noted for the pure PHBV. This suggests that the material is more elastic than others. For the “0” sample, the curve is characterized by a higher elastic modulus than for other cycles. However, the lower value of the maximum stress and much lower elongation were noted. The decrease in the maximum stress and the increase in stiffness were also observed with each subsequent recycling cycle.

Some characteristic differences and similarities caused by the form of the additive and the method of processing can be noticed on the basis of the literature review concerning biocomposites and composites with natural fibers as a filler [54,55,56,57,58,59]. In this work, the Young elastic modulus for GBH–PHBV biocomposites was from 3620.52 MPa (“0” sample) to 4147.43 MPa (“5x” sample). It confirms the significant increase in the stiffness after the addition of buckwheat hulls and after the subsequent cycles. In the literature [74,75,76], the elastic stiffness of PHBV with different natural fillers, e.g., with rice husk, hemp fibers, and flax, is in the range of 3000–4000 MPa, which is consistent with the results of this study. The application of GBHs in the form of fine powder promotes better integration to the polymer, which leads to an increase in the stiffness and the elastic modulus, especially after the subsequent recycling cycles. 

The results of the tensile strength are also similar to those obtained by other researchers for biopolymers with different natural fillers [52,53,54]. The tensile strength in the range of 20–25 MPa was noted for the composite with rice husk [77]. In [52,62,78], the tensile strength for the PHBV with hemp or flax fibers was in the range of 30–40 MPa, and it was higher for results obtained here. The lower strength of the material tested here is probably caused by a lack of long fibers that can act as the reinforcement. The presence of natural fibers in biocomposite, due to their length and strength, transfers stresses in the material more effectively than non-fiber fillers, which results in the increase in the tensile strength values. For the biocomposite tested here, the application of GBHs in powder form limits this effect and, therefore, the lower tensile strength values are achieved.

The elongation at break was in the range of 1.30–1.70% for the biopolymer composite and was 4.12% for the pure PHBV. It was in the range of 1–7% for composites with natural fibers or with larger particles of hulls [53,79,80]. The lower values of elongation for the biocomposite tested are related to the more brittle behaviour of the material, which is characteristic for composites with fine powder particles. The analysis of the literature shows that the addition of fine particles of hulls or other mineral fillers often leads to the decrease in the elongation at break in the wake of the limitation of chain mobility [36,81].

The degradation of the mechanical properties of biocomposites, especially the tensile strength and the elongation, is often observed during the subsequent recycling cycles [82,83]. The results indicate the stabilization of parameters after the first recycling cycle, which might be a result of the improvement of the microstructure after the reprocessing. In the literature [82,83,84], similar observations are noted mainly for composites with fine fillers, which indicate better particles embedding in a polymer matrix after the recycling process. They are also characterized by the increased homogeneity, which might compensate for the degradation of mechanical properties [84,85]. Additionally, the mechanical degradation is very clear for composites with larger particles or with natural fibers after the recycling. It can be explained by the damage of fibers or by the lower adhesion between phases [67].

To compare results obtained with the literature, the GBH–PHBV biopolymer composite is charaterized by the higher elastic modulus, as well as by the lower tensile strength and the elongation at break. The application of fine powder particles contributes to their better integration with the polymer matrix, which increases the stiffness of the material but at the same time limits its flexibility. The results obtained after the recycling cycles also indicate the stabilization of mechanical properties, which is consistent with observations for another biocomposites with fine particle fillers but less common for composites with fibers.

**Figure 6:**
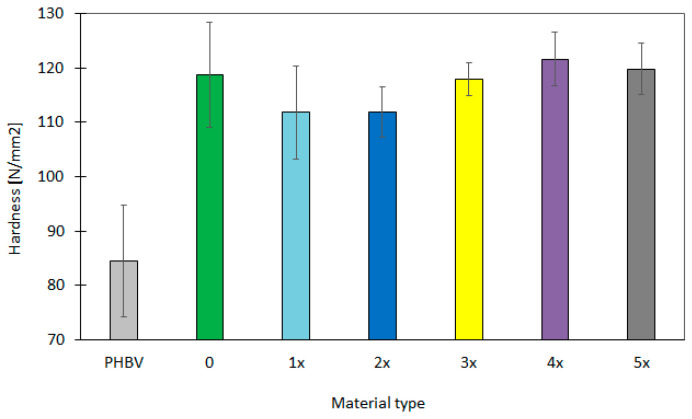
The graphical representation of hardness in four areas for composite originally produced (0 sample) and after the subsequent recycling cycles (1x–5x).

The hardness of GBH–PHBV biocomposites for originally produced samples, as well as after the recycling cycles, was also determined. The hardness was measured in nine selected points along the sample. The results with their statistical analysis are contained in Table 4. The results show the significant differences in the hardness distribution (Figure 6), which might be caused by the structural changes in the biocomposite in the wake of the recycling process. It can be also a result of differences in the deposition of the powder particles of buckwheat hulls.

For the “0” sample, the hardness was in the range of 108.9–124.5 N/mm^2^, with an average value of 115.4 N/mm^2^. The standard deviation had a relatively high value, especially for measuring points (4–6 points) in the center of the sample. The variation coefficient reached the maximum value of 0.11 at point 8, which is problably related to the irregular embedding of powder particles on the surface layer of the sample. After the first recycling cycle (1x), the average value of hardness decreased to 111.9–123.0 N/mm^2^. The reduction in the standard deviation was also noted. The variation coefficient decreased for most measurement points. This suggests improvement in the repeatability of the results and the homogeneity of the composite microstructure after the first processing cycle. In the second recycling cycle, the hardness increased slightly in comparison to the first cycle. The further reduction in the standard deviation was also noted. The variation coefficient was in the range of 0.02–0.05 for the second recycling cycle. During the third recycling cycle (3x), the hardness values increased and reached a maximum value of 131.5 N/mm^2^ at point 5. The standard deviation decreased for all measurement points and the coefficient of variation did not exceed 0.04. This indicates further improvement in the uniformity and stability of mechanical properties. In the fourth cycle (4x), the hardness values were slightly higher and reached average values in the range of 121.6–134.8 N/mm^2^. The variation coefficient reached the lowest values in the fourth cycle, which proves the maximum homogeneity of the material. The hardness was the highest for the last tested cycle (5x), and it reached the values in the range of 119.1–139.1 N/mm^2^. The standard deviation also decreased, reaching the lowest values among all cycles. This might be related to the optimal deposition of buckwheat hull particles in the polymer matrix. The variation coefficient reached low values, which confirms the high repeatability of the results and the dimensional stability of the molders after repeated processing. In contrast, the pure PHBV biopolymer is characterized by the average hardness values of 74.0–84.5 N/mm^2^, with a high standard deviation (10.2–14.2 N/mm^2^). This might confirm the higher variablity of results and less uniform structure of the material. Additionally, the variation coefficient was relatively high (to up 0.18) for the pure PHBV, which suggests that the material had less stable mechanical properties in different points of the sample. The lower hardness vaue for the pure PHBV, compared to the biopolymer composite tested, indicates the greater flexibility. The strengthening effect that is characteristic for the application of GBHs was not also noted for the pure PHBV biopolymer.

The analysis of hardness for the GBH–PHBV biopolymer composites also showed the significant changes in the hardness distribution for different recycling cycles. Firstly, the greater variablity of results and the higher standard deviation was observed, which is caused by the irregular embedding of GBHs on the surface of the sample originally produced. The variability of results decreased with subsequent processing cycles in the wake of the improvement of the microstructural homogeneity. The optimal embedding of powder particles in the polymer matrix, as well as the better adhesion after repeated processing, can lead to stabilization of mechanical properties and an increase in the hardness for molders. This can be essential from the point of view of the material’s potential applications.

**Figure 7:**
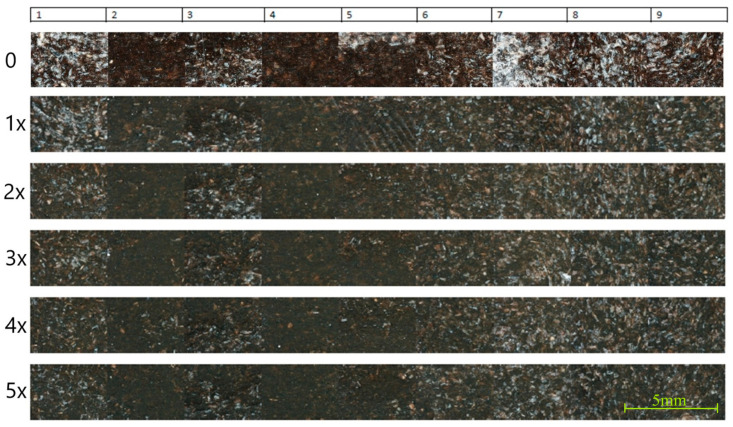
Photographs of representative probes for the composite originally produced (“0”) and for ones after the subsequent recycling cycles (1x–5x) in particular measurement areas.

The photographs of the surface layer of molders at particular measurement points are presented in Figure 7. The analysis of the microstructure of GBHs in the PHBV matrix was conducted. The high amount of buckwheat hulls with irregular distribution is visible on the surface of the “0” sample. The particles with greater size are clearly observed. The chaotic arrangement of GBHs was noted particularly in the center parts of the sample (4–6 areas). The structure can cause local irregularities that might affect the shrinkage and the mechanical properties. The small areas of polymer matrix surrounding the buckwheat hulls were detected, which might limit their full adhesion to the polymer. 

After the first recycling cycle, the GBH microstructure is more homogeneous. It can be seen that the fine particles of GBH are better embedded in the polymer matrix. Their protrusions on the surface are smaller compared to those of the sample originally produced. In measurement points located in the center of the sample, GBHs are more embedded in the structure of the material, which is a result of better wetting of the powder by the polymer during reprocessing. This change might influence the increase in the dimensional stability and the improvement of transverse and longitudinal shrinkage. Further microstructure alignment was noted during the second recycling cycle. The GBH particles are less visible on the surface, which might indicate their deeper embedding in the polymer. The even distribution of GBH particles is visible in measurement points, and the polymer matrix is more homogeneous. This structure might help reduce the shrinkage in horizontal planes, which was also observed in the shrinkage analysis.

The layer is more homogeneous after the third recycling cycle. The GBHs are largely integrated into the polymer matrix, and their visibility on the surface is limited. The measurement areas show smaller differences in the deposition of GBHs in comparison to previous samples, which might indicate greater stability of the microstructure after subsequent recycling cycles. This homogenization of the structure can lead to further stabilization of shrinkage and mechanical properties.

In the “4x” sample, the surface structure becomes smoother and GBHs are almost completely surrounded by the polymer matrix. The microstructure is very homogeneous, and differences between individual measurement areas are minimal. The embedding of GBHs in the matrix is so effective that they become part of the integral structure of the material. It improves the uniformity of mechanical properties and the stability of volume shrinkage.

The sample surface is the most uniform for the fifth recycling cycle, and the hulls are fully embedded in the polymer. The measurement areas do not show significant differences in microstructure, which indicates the maximum stabilization of the structure after the repeated processing. This deep integration of GBHs in the polymer matrix might be associated with the reduction in transverse and thickness shrinkage. The GBHs are evenly spaced and act as a reinforcement that stabilizes the polymer.

As can be seen, the addition of GBHs in the powder form and their embedding in the composite structure is more and more homogeneous with the subsequent recycling cycles. Initially, larger scale particles visible on the surface are gradually integrated into the matrix, leading to homogenization of the microstructure. This can influence the mechanical properties and can improve the shrinkage repeatability. The recycling process also increases the homogeneity of properties in different measurement areas.

The analysis of the microstructure of GBH–PHBV biocomposites presents the significant differences caused by the use of fine powder instead of full or partially crushed hulls. The powdered form of the hulls promotes their even distribution in the polymer matrix. This results in better embedding of particles in the composite structure, especially after several processing cycles. This distribution ensures better deposition of particles on the surface of samples, which reduces the occurrence of local stress concentrations and potential defects that can occur for coarser particles of the filler. The fine powder particles have a larger contact surface with the polymer, which improves adhesion at the interface and ensures the dimensional stability of the entire composite. This phenomenon might be observed as the stabilization of shrinkage parameters in subsequent processing cycles. 

In comparison to publications in which buckwheat hulls are applied in the form of coarser particles or even as uncrushed ones, the microstructure of composites with the powder filler has significantly greater uniformity [86,87,88]. The results of a study for coarser particles of the fillers show the occurrence of the clear and irregular appearance of scales on the composite surface. This might result in uneven shrinkage and larger deviations in dimensions after processing. Additionally, the coarser particles create the local clusters, and they can introduce higher stresses into the matrix, which negatively affects the uniformity of the structure and mechanical stability, especially with regard to the volume shrinkage.

The literature review concerns the composites and biocomposites with coarser particles, as the filler often shows the problem associated with the full embedding of these particles into the polymer [79,80,86,87,89]. This results in difficulties with the full integration and, therefore, the application of higher amounts of plasticizers, or adhesion-improving additives are needed. For fine particles, such additives might be not necessary because they create strong bonds with the polymer on a microscale. It is observed, e.g., in the GBH–PHBV biocomposite polymers tested here.

The analysis of hardness and the surface microstructure of samples produced from GBH–PHBV biocomposite shows the correlation between the powder particles and the hardness in different recycling cycles. For the “0” sample, the surface is most uneven, and the particles are visible as large, irregular fragments. This leads to significant differences in hardness between particular measurement points. Such uneven distribution of hulls and their large size might cause local weakness in the structure.

The microstructure is more homogeneous for samples after the first recycling cycle (1x). This is visible as the smoother surface with better-embedded GBH particles in the photographs, and it explains the decrease in the standard deviation and more even hardness values. The better wetting and deeper embedding of fine particles might also promote the improvement of the adhesion between particular phases. This leads to the increase in the uniformity of mechanical properties. The microstructure is more and more homogeneous for further recycling cycles, especially for the second and the third ones. The surface of samples is smoother, and a smaller amount of hull particles is visible on the surface layer. This correlates directly with the increase in the hardness and with the decrease in the variation coefficient. The recycling process promotes further fragmentation and better embedding of GBHs, which can decrease the stress concentration and limit the occurrence of local defects on the surface of samples.

The highest hardness values are observed for samples after the fourth (4x) and the fifth (5x) recycling cycles. The photographs of the microstructure also show the almost-complete embedding of GBH particles in the polymer matrix. The homogenization of the microstructure results in minimal differences in hardness between particular measurement points. This is also confirmed by the low values of the standard deviation and the variation coefficient. The fine powder particles are fully integrated with the polymer, and they create a more compact and stable structure.

## 4. Summary and Conclusions

This paper presents the possibility of the application of ground buckwheat hulls (GBHs) in the powder form as a filler in biocomposites. The analysis of selected mechanical and functional properties of biocomposites was conducted. The influence of the repeated processing of the biocomposite on mechanical and functional properties was also examined. The main conclusions are as follows:

1. The reduction in shrinkage in all directions was noted in comparison to the pure PHBV. This might indicate that the filler acts as the dimensional stabilizer. The powder form of the GBHs allowed for even distribution of particles in the matrix, which has reduced the possibility of material deformation during the cooling process and has limited the shrinkage.
2. The GBH–PHBV biocomposites showed much greater water adsorption in contrast to the clear PHBV. The increased ability to absorb water in the biocomposite is directly related to the presence of hydrophilic particles of GBH containing cellulose and lignin.
3. The multiple processing has led to the structural degradation of the polymer characterized by the reduction in the tensile strength and the elongation at break.
4. The initial decrease in the tensile impact strength was noted during the recycling process. But then the parameter has stabilized at the level of 5.92 kJ/m^2^, showing the improvement of the microstructure homogeneity and the better embedding of the filler particles.
5. The addition of GBHs has also influenced the increase in the biocomposite hardness compared to the pure PHBV. The parameter increased with the subsequent recycling cycles, which might be an effect of better integration of filler particles with the matrix and the reduction in porosity.
6. The microscopic analysis shows that the surface of samples has changed with the subsequent processing cycles. After several cycles (3x–5x), the particles were embedded deeper and the surface was more homogenous. A more homogenous surface of the material was observed after the recycling process.
7. The recycling process decreased the mechanical properties initially, but the stabilization of the microstructure and the improvement of the composite homogeneity were observed after several processing cycles.
8. The results show that GBH–PHBV biocomposite might be a more dimensionally stable and more recycling resistant alternative to pure polymers. This is particularly important in terms of the sustainable development and the reuse of materials in industry sectors.

In further research, the FTIR, DSC, TGA and SEM analyses will be conducted in order to correlate the mechanical properties with the chemical changes within the material.

## Figures and Tables

**Table 1:** Technological parameters of the injection molding of probes dedicated to the uniaxial tensile tests.

Parameter	Value
Mold temperature (°C)	90
Melt temperature (°C)	190
Cooling time (s)	25
Packing time (s)	25
Packing pressure (MPa)	55
Injection speed (cm^3^/s)	35

**Table 2:** Shrinkage for samples with the statistical analysis.

Material Type	Parameter	Shrinkage			
		Longitudinal	Transverse	In Thickness	Volumetric
PHBV	x [%] *	2.50	2.7	4.85	10.77
	s *	0.66	0.6	0.92	0.8712
	V *	0.2640	0.2222	0.1897	0.8089
0	x [%]	1.07	1.35	0.62	3.02
	s	0.0142	0.0506	0.1244	0.1270
	V	0.0132	0.0374	0.2010	0.0421
1x	x [%]	1.22	1.91	0.93	4.01
	s	0.0253	0.1420	0.1983	0.3204
	V	0.0208	0.0743	0.2128	0.0800
2x	x [%]	1.21	1.90	0.93	3.99
	s	0.0213	0.1033	0.1163	0.2042
	V	0.0176	0.0543	0.1249	0.0511
3x	x [%]	1.21	1.92	0.98	4.06
	s	0.0120	0.0620	0.0762	0.0777
	V	0.0099	0.0322	0.0777	0.0192
	x [%]	1.23	1.96	1.00	4.14
4x	s	0.0149	0.0685	0.0000	0.0691
	V	0.0121	0.0349	0.0000	0.0167
	x [%]	1.21	1.91	1.05	4.12
5x	s	0.0181	0.0723	0.1016	0.1107
	V	0.0149	0.0378	0.0965	0.0268

* x—the average shrinkage, * s—standard deviation, * V—variation coefficient.

**Table 3:** Results obtained in uniaxial tension tests for the clear PHBV biopolymer and for GBH–PHBV biopolymer composites (samples 0–5x).

Material	Parameter	E [MPa]	σ_M_ [MPa]	ε_M_ [%]
PHBV	x [%]	2617.37	35.48	4.12
	s	112.02	0.86	0.15
	V	4.28	2.42	3.63
0	x [%]	3620.52	24.50	1.70
	s	99.28	0.31	0.035
	V	2.74	1.27	2.063
1x	x [%]	4028.94	20.57	1.30
	s	84.21	0.25	0.032
	V	2.09	1.22	2.457
2x	x [%]	3944.50	19.96	1.35
	s	80.11	0.21	0.029
	V	2.03	1.05	2.142
3x	x [%]	3941.35	20.08	1.36
	s	79.57	0.19	0.027
	V	2.02	0.95	1.990
	x [%]	3998.18	20.37	1.33
4x	s	78.86	0.19	0.025
	V	1.97	0.93	1.878
	x [%]	4147.43	20.42	1.37
5x	s	79.69	0.20	0.025
	V	1.92	0.98	1.820

**Table 4:** Hardness of samples (0–5x) in conjunction with the statistical analysis of results.

Material	Parameter	Measurement Point								
		1	2	3	4	5	6	7	8	9
PHBV	x [N/mm^2^]	77.8	74.0	79.6	84.5	82.6	76.4	74.7	78.2	79.4
	s	12.6	13.1	11.9	10.2	11.6	12.4	13.5	14.2	13.9
	V	0.16	0.18	0.15	0.12	0.14	0.16	0.18	0.18	0.18
0	x [N/mm^2^]	122.4	108.9	115.3	118.7	119.7	109.7	108.6	110.8	115.4
	s	7.9	6.5	7.8	9.7	8.9	7.4	8.2	12.4	12.9
	V	0.06	0.06	0.07	0.08	0.07	0.07	0.08	0.11	0.11
1x	x [N/mm^2^]	115.0	114.4	109.5	111.8	121.0	119.6	119.9	120.1	123.0
	s	6.2	6.3	3.6	8.5	5.2	5.8	6.9	6.6	6.3
	V	0.05	0.06	0.03	0.08	0.04	0.05	0.06	0.06	0.05
2x	x [N/mm^2^]	117.5	110.7	114.0	111.9	127.7	122.3	126.2	119.1	128.4
	s	4.1	4.6	5.4	4.6	3.1	5.7	4.1	3.2	3.8
	V	0.04	0.04	0.05	0.04	0.02	0.05	0.03	0.03	0.03
3x	x [N/mm^2^]	115.0	111.7	114.2	117.9	128.4	125.1	129.5	123.5	128.6
	s	4.1	6.7	4.8	3.1	6.5	2.3	4.8	5.3	3.8
	V	0.04	0.06	0.04	0.03	0.05	0.02	0.04	0.04	0.03
	x [N/mm^2^]	118.7	121.3	118.0	121.6	132.1	131.1	131.0	128.8	134.8
4x	s	4.0	6.1	4.7	4.9	2.6	4.3	3.6	3.5	2.9
	V	0.03	0.05	0.04	0.04	0.02	0.03	0.03	0.03	0.02
	x [N/mm^2^]	119.1	132.5	121.6	119.8	135.0	135.9	131.8	133.9	139.1
5x	s	2.1	8.1	3.9	4.7	3.1	5.8	6.2	3.2	3.7
	V	0.02	0.06	0.03	0.04	0.02	0.04	0.05	0.02	0.03

## Data Availability

Data are contained within the article.
